# Effects of intensity, attention and medication on auditory-evoked potentials in patients with fibromyalgia

**DOI:** 10.1038/s41598-020-78377-0

**Published:** 2020-12-14

**Authors:** N. Samartin-Veiga, A. J. González-Villar, Y. Triñanes, C. Gómez-Perretta, M. T. Carrillo-de-la-Peña

**Affiliations:** 1 Departamento de Psicoloxía Clínica e Psicobioloxía, Facultade de Psicoloxía, Universidade de Santiago de Compostela, Santiago de Compostela, Spain; 2 Psychological Neuroscience Lab, Psychology Research Centre, School of Psychology, University of Minho, Braga, Portugal; 3 Research Foundation of La Fe Hospital, Valencia, Spain

**Keywords:** Cognitive neuroscience, Sensory processing

## Abstract

Fibromyalgia (FM) has been associated to an increased processing of somatosensory stimuli, but its generalization to other sensory modalities is under discussion. To clarify this, we studied auditory event-related potentials (AEPs) to stimuli of different intensity in patients with FM and healthy controls (HCs), considering the effects of attention mechanisms and medication. We performed two experiments: In study 1 (n = 50 FM, 60 HCs), the stimuli were presented randomly within the sequence; in study 2 (n = 28 FM, 30 HCs), they were presented in blocks of the same intensity. We analyzed intensity and group effects on N1-P2 amplitude and, only for the FM group, the effect of medication and the correlation between AEPs and clinical variables. Contrary to the expectation, the patients showed a trend of reduced AEPs to the loudest tones (study 1) or no significant differences with the HCs (study 2). Medication with central effects significantly reduced AEPs, while no significant relationships between the N1-P2 amplitude/intensity function and patients’ symptoms were observed. The findings do not provide evidence of augmented auditory processing in FM. Nevertheless, given the observed effect of medication, the role of sensory amplification as an underlying pathophysiological mechanism in fibromyalgia cannot be discarded.

## Introduction

Fibromyalgia (FM) is a complex chronic syndrome that affects around 5% of the population, producing a remarkable burden on their quality of life^1^. FM is mainly characterized by widespread pain, constant tiredness and fatigue, cognitive dysfunction, and non-restorative sleep. Despite extensive research, the etiology and pathogenesis of FM remain unclear. Several studies postulate that the patients with FM present dysfunction of central nervous system mechanisms, affecting both nociceptive and non-nociceptive processing^2–5^, and including alteration in the descending pain modulation mechanisms^3,6^.

Based on those evidences, it has been proposed that central amplification of painful and non-painful sensory inputs may be at the basis of this syndrome, as well as of other chronic pain diseases^7^. For FM, this hypothesis has received consistent support in the somatosensory modality: patients show increased pain scores and less tolerance to noxious stimuli^8–12^ as well as alterations in electroencephalography (EEG) activity and deficits in habituation while processing somatosensory stimuli (painful and non-painful)^13–18^. However, although patients with FM report increased sensitivity to olfactory, auditory and, visual stimuli^19,20^, the evidence supporting the generalization of this pattern of central amplification to modalities other than somatosensory is not conclusive.

In the auditory modality, several studies reported augmented subjective perception of tones intensity and lower tolerance to noise by FM patients^19,21^. Also, using a validated paradigm,—the loudness dependence of the Auditory Evoked Potentials (AEPs)—, Carrillo-de-la-Peña^22^ found greater amplitude of the brain electrical response evoked by intense auditory stimuli in FM patients. In contrast, other authors did not find differences in auditory cortical responses between the FM and the control groups^15,23,24^. These inconsistencies in the literature may be due to differences in paradigms and stimulation parameters. For instance, some studies employed a paired click paradigm to analyze preattentional components^15,24^, while others analyze later brain responses^22,23^. In addition, some authors argue that the inconsistent results may be due to the stimuli intensity, sometimes not powerful enough to be unpleasant and to elicit changes in information processing^25^. However, this suggestion goes against the hypothesis of a generalized hypervigilance in FM, which should be present to both nociceptive and harmless stimuli^26,27^.

Pain perception and attention proccesses share neural substrates^28,29^. Different modes of attention to painful stimuli (bottom-up vs. top-down) differentially modulate the evoked brain response^30,31^. Since the presentation of auditory stimuli in blocks or in a random sequence may engage different attentional procesess^32,33^, it would be interesting to analyze the effect of attention on AEPs recorded while processing tones of increasing intensity in a sample of patients with FM, as compared to healthy controls. Also, as previous studies suggest that the effect of medication may explain, at least partially, the inconsistencies in the literature^24,34^, the control of this variable is of paramount importance.

Therefore, the aim of the present paper was to analyze the brain electrical activity to auditory stimuli varying in intensity, and whether that response depend on the attentional mechanism involved, in a sample of patients with FM and healthy controls. To this end, we recorded AEPs to two tones sequences: random presentation (study 1), where the subject could not anticipate the appearance of the louder stimuli (involving bottom-up mechanisms); and presentation in blocks of equal intensity (study 2), where the subject could anticipate the intensity of the following stimuli (involving top-dow mechanims). According to the increased sensory processing hypothesized for FM, we expected to find larger AEPs to the more intense tones in the patients, specially when they are predictable, as in the blocks presentation.

To control for the effect of medication, we compared patients using or not mediation with central nervous system (CNS) effects (antidepressive, anxiolytic, anticonvulsivant, etc.). Furthermore, since the intensity dependence of the AEPs has been proposed as an indicator of the serotonin function^35–38^, linked to pain inhibition^39,40^, and mood regulation^41^, we expected to find a positive correlation between the intensity/amplitude function of the AEPs, and FM symptoms’ severity.

## Methods

### Participants

For study 1, we recruited 50 patients with FM (mean age: 47.30; SD: 8.00) and 60 healthy controls (HC) (mean age: 45.06; SD: 10.34) and for study 2, 28 patients with FM (mean age: 50.31; SD: 9.20) and 30 HC (mean age: 48.44; SD: 11.18). All the participants were female and both groups were matched for age in each study. The patients with FM had a previous diagnosis confirmed by their primary care physician or rheumatologist and had no other disease that could explain chronic pain nor mental disorders (except mild/moderate levels of depression, or anxiety). The same exclusion criteria were applied to the participants of the control group, who also should not have chronic pain problems. Additional exclusion criteria for both groups comprised a history of substance abuse or brain damage.

The diagnosis of participants was confirmed using the 1990 ACR criteria^42^ and/or the 2010 ACR criteria^43,44^. Moreover, in both studies, the participants were evaluated though a comprehensive clinical interview and several self-reported questionnaires (see “Clinical assessment”). Also, only in study 1, we assessed the 18 tender points by pressure algometry. Written informed consent was obtained from all the participants and they were coded by numbers. The studies were approved by the Ethics Committee of the University of Santiago de Compostela and were executed in accordance with the Code of Ethics of the Declaration of the World Medical Association in Helsinki, 2013.

### Clinical assessment

#### Study 1

Participants completed several self-reported questionnaires for the assessment of FM symptoms (all in their validated Spanish versions).

##### Visual-analogue scales (VAS)

They were created ad-hoc to evaluate the clinical status of participants. The scales consist of a set of horizontal 10 cm-long lines where participants had to indicate their status in the following variables: pain, health status, mood status, and non-restorative sleep. All the scales were presented so that the left end indicated the best condition, and the right the worst.

##### Fibromyalgia impact questionnaire (FIQ)^45,46^

The FIQ is a self-reported questionnaire of 10 items to evaluate the health status of patients with FM. It includes indices of functionality as well as the core symptoms of fibromyalgia, with a maximum score of 100 (higher scores are associated to more severe symptoms).

##### SF-36 health survey^47,48^

The SF-36 is a questionnaire that assesses quality of life. It is made up of 36 items that cover 8 dimensions (physical functioning, role physical, bodily pain, general health, vitality, social functioning, role emotional, and mental health) and provide a profile of health status and functionality. For each dimension, the items are coded, added and transformed into a range of 0–100 (from the worst health status to the best health status). We computed a mean of the 8 dimensions to obtain a general index of quality of life (SF-36 general).

##### Beck depression inventory (BDI)^49,50^

The BDI evaluates depressive symptomatology using 21 items scored from 0 to 3 (the higher the score, the higher the severity of depressive symptoms).

##### The pittsburgh sleep quality index (PSQI)^51,52^

The Spanish validation of the PSQI was used to assess the quality and dysfunction of sleep in the last month. It is composed of 7 subscales (from 0 -no difficulty- to 3 -maximum difficulty-) that describe different aspects of sleep problems. The overall score has an interval of 0–21, where the highest scores indicate a worse quality of sleep.

##### Algometry

We measured the pain threshold and tolerance at the 18 tender points using a pressure algometer (Wagner Force One, Model FDI; Wagner Instruments, Greenwich, CT, USA). Pressure pain threshold was defined as the minimum force applied that induces pain, and pressure pain tolerance as the maximum pain-pressure value that was bearable. These measures were quantified at each of the 18 specific tender point sites according to the ACR 1990 criteria for FM. A tender point was considered positive when the patient felt pain at pressures equal or less than 4 kg/cm^2^. For each participant we calculated the total count of positive tender points and the mean pain-pressure threshold and tolerance at the 18 points.

#### Study 2

As in study 1, the participants completed several self-reported questionnaires (including VAS, BDI, and PSQI). To assess the diagnostic criteria proposed by Wolfe^43^, we used the Spanish version of the Fibromyalgia Survey Questionnaire (FSQ)^44^ originally published by Wolfe^53^. It includes the Symptom Severity Scale (SSS), which considers 3 key symptoms (fatigue, cognitive problems (attention, concentration, or memory), and non-restorative sleep) and others such as abdominal pain, depression, and headache; and the Widespread Pain Index (WPI) that indicates the number of body areas with pain reported by the patient.

### Procedure and task

Participants were asked to not smoke, consume coffee, alcohol, or other drugs not medically prescribed before evaluation. At first, participants completed the clinical interview and self-reported questionnaires. In study 1, the 18 tender points (ACR, 1990) were evaluated in patients and control participants using a pressure algometer. In both experiments the participants were seated in a comfortable chair in a room isolated from external sounds and with dim light. After that, participants were fitted with an electrode cap to register their EEG.

In study 1, before the recording, the audiological status of both ears was verified by evaluation of the auditory threshold (Békésy's method). Four patients with FM and one healthy control were discarded due to their high thresholds in the audiometry. Also, one patient with FM was eliminated due to technical problems during the EEG recording. AEPS were obtained using a pseudo-randomized series of 288 auditory stimuli, 72 of each intensity (S1 = 70, S2 = 80, S3 = 90; and S4 = 105 dB SPL). The stimuli had a frequency of 1000 Hz and a duration of 50 ms, with a random interstimuli interval of 1500 ± 100 ms. Each intensity was equally preceded by each one of the other 3 intensities.

In study 2, two patients with FM were discarded due to hearing impairment and complains of annoyance by the loudest tones during the recording, respectively. The task consisted of 180 trials, 60 of each intensity (S1 = 70, S2 = 90; and S3 = 105 dB SPL), presented in blocks of equal intensity. The stimuli had a frequency of 1000 Hz and a duration of 50 ms, with a random interstimuli interval of 1400 ± 100 ms. There were two forms of stimuli presentation (increasing or decreasing intensity) which were counterbalanced across participants.

### Recording and processing of AEPs

Brain electrical activity was recorded during the presentation of the sounds. In the study 1, the EEG was recorded using a 32-electrocap (Electro-cap International, Inc., Eaton, OH, USA) and a Synamps amplifier (Neuroscan Labs, Charlotte, NC, USA) for EEG collection, amplification and online filtering. In the study 2, an ActiChamp system with 32 electrodes cap (Brain Products inc.) was used. The electrodes were placed according to the 10-20 International System, with the reference electrode in the tip of the nose. The ground electrode was placed in the Fpz position. Additional electrodes were placed 1 cm above and below the eyes and other two were placed on the outer edge of each eye to correct vertical and horizontal eye movements. The EEG was digitized at 500 Hz, 10,000× amplified, and filtered with a 0.1–100 Hz band pass filter and a 50 Hz notch filter. Impedances were kept below 10 KΩ.

EEG data were analyzed using the EEGlab 13.3 toolbox^54^. The EEG was re-referenced to an average reference. A spherical-spline interpolation was used to reconstruct bad channels. EEG noisy segments, produced by eye movements or other contaminants, were rejected by visual inspection. The data were digitally filtered using a 0.5 Hz high pass filter and a 30 Hz low pass filter. Epochs were extracted from 0.4 s pre-stimulus to 1.2 s post-stimulus. An extended Independent Component Analysis algorithm (ICA) was applied to the electrophysiological data^55^, and components related to ocular or muscular activity were removed after visual inspection.

The EEG epochs used for the analysis of AEPs were corrected using a baseline from − 0.2 to 0 s. We measured N1 and P2 peak amplitudes at each stimuli intensity in electrodes Fz and Cz, given that these are the locations where such components usually show their greatest amplitudes^32^. In study 1, the N1 component was identified as the most negative peak within the range 50–160 ms in Fz, and within the range 90–160 ms in Cz; and P2 as the most positive peak within the range 150–300 ms in Fz and Cz. In study 2, N1 was identified as the most negative peak within the range 80–150 ms in Fz and Cz; and P2 as the most positive peak within the range 150–300 ms in Fz, and within the range 160–300 ms in Cz. The amplitude of N1-P2 was calculated as the peak-to-peak difference.

### Data analyses

Student t-tests were performed to evaluate possible differences between groups in demographic and clinical variables. Also we calculated the percentages of patients using each medication pattern (classified by: analgesics, nonsteroidal anti-inflammatory drugs (NSAIDs), anxiolytics, opioids, migraine medication, antidepressants, sedatives, and antiepileptic drugs). Repeated measures ANOVAs with Electrode (Fz vs. Cz) and Intensity (70, 80, 90, 105 dB; or 70, 90, 105 dB, for study 1 and 2, respectively) as intra-subject factors and Group (FM vs. HCs) as a between-subject factor were conducted for N1-P2 amplitude in each study. We adjusted the degrees of freedom by the Greenhouse–Geisser correction to determine the significance levels when the sphericity assumption was violated. For patients with FM, we additionally performed a mixed model ANOVA with Electrode and Intensity as intra-subject factors and Medication Group (with vs. without central medication) as a between-subject factor on N1-P2 amplitude. Effect size analyses were undertaken to assess the magnitude of differences between the groups (Cohen’s d). Finally, Pearson’s correlation analyses were performed to evaluate the relation between psychophysiological and clinical variables; for this purpose, we first calculated the amplitude/intensity slope of the N1-P2 by linear regression for each subject, as a measure of the intensity dependence of the AEPs. Then, we calculated the correlations with the clinical indices, for the total FM group, and separately for each group of medication. All these statistical analyses were performed with SPSS (v. 20; http://www.ibm.com). Significance level was *p* value < 0.05.

## Results

### Study 1

#### Demographic and clinical variables

As may be seen in Table 1, t-tests confirmed that both groups were comparable in age. Patients showed significantly more affectation in all the FM symptoms, as well as lower pressure pain threshold and tolerance values, with large effect sizes (Cohen’s d > 0.80) [^56^; pp. 280–287]. Although all the patients were previously diagnosed according to the ACR recommendations^42^, we found that 5 of them did not fulfill the criterion of having more than 11 tender points with values lower than 4 kg/cm^2^. Due to the criticisms around this criterion^57^, we decided to retain those participants for the analyses. The medication pattern of the patients was the following: 24% of them used NSAIDs, 28% anxiolytics, 30% antidepressants and 6% antiepileptic (see *Supplementary material*; Supplementary Table S1).

**Table 1:** Demographic and clinical variables for patients with fibromyalgia (FM) and healthy controls (HC). Results of the t-tests comparisons and effect sizes (Cohen’s d).

Variable	FM n	FM Mean (SD)	HC n	HC Mean (SD)	t	P	Cohen’s d
Age	50	47.30 (8.00)	60	45.06 (10.34)	1.27	n.s	–
Years of education	49	16.94 (4.32)	56	19.11 (4.56)	− 2.49	< 0.05	0.20
VAS pain	49	6.82 (1.99)	57	1.83 (2.14)	12.35	< 0.001	2.41
VAS health status	50	6.82 (2.20)	57	1.87 (2.50)	10.79	< 0.001	2.10
VAS mood status	50	4.45 (2.81)	57	1.69 (2.01)	5.77	< 0.001	1.13
VAS non-restorative sleep	50	7.24 (2.72)	57	2.73 (2.92)	8.24	< 0.001	1.60
FIQ total	50	64.51 (15.67)	–	–	–	–	–
SF36 general	49	41.16 (14.16)	57	79.80 (11.70)	− 15.38	< 0.001	2.97
BDI	50	19.22 (9.43)	58	5.57 (5.93)	8.84	< 0.001	1.73
PSQI total	49	12.63 (4.44)	56	5.21 (3.58)	9.47	< 0.001	1.84
Tolerance (algometry)	49	3.21 (1.04)	58	6.11 (1.02)	− 14.49	< 0.001	2.82
Threshold (algometry)	49	2.42 (0.82)	58	5.55 (0.99)	− 17.61	< 0.001	3.44

VAS: Visual Analogue Scale; FIQ: Fibromyalgia Impact Questionnaire; BDI: Beck Depression Inventory; PSQI: Pittsburgh Sleep Quality Index; n.s.: non-significant.

#### AEPs to tones

**Figure 1:**
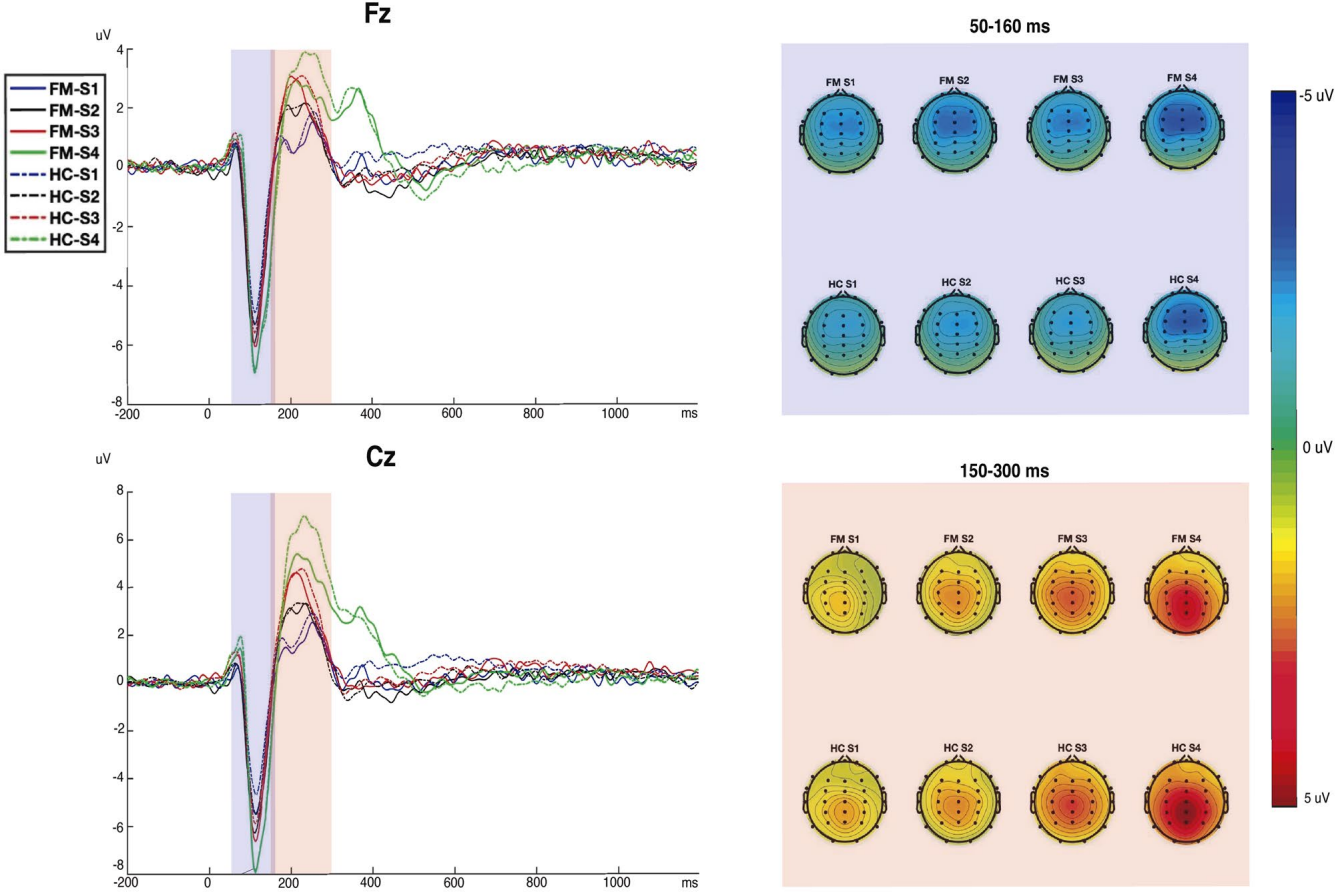
Right. Electrical brain activity in response to auditory stimulation in patients with fibromyalgia (FM) and healthy controls (HC). The waveforms represent the auditory evoked potentials (AEPs) at electrodes Fz (up) and Cz (bottom) elicited by the tones of 70 dB (S1; blue line), 80 dB (S2; black line), 90 dB (S3; red line) and, 105 dB (S4; green line). Left. Spatial distribution of the electrical activity elicited by stimuli of different intensities. The upper part shows the topographic representation of N1 (measured as the mean value from 50 to 160 ms after the presentation of the stimuli), and the lower part shows the topography of P2 (measured from 150 to 300 ms).

Figure 1 (random presentation) shows larger N1-P2 amplitudes at Cz than Fz, and linear amplitude increases as intensity increased, as well as differences between both groups only for the most intense tones. Also, it can be seen that the topographical distribution of N1 was fronto-central, while the distribution of P2 was centro-parietal in both groups.

A repeated measures ANOVA on N1-P2 amplitude showed a significant effect of Electrode [F_(1,102)=_143.11; p < 0.001; Cohen’s d = 2.35], Intensity [F_(3,306)_ = 141.82; p < 0.001; Cohen’s d = 2.35], and Electrode × Intensity [F_(3,306_) = 78.33; p < 0.001; Cohen’s d = 1.73]. The amplitudes increased as the stimuli intensity increased, being larger at Cz than Fz, specially for the highest intensities. Post-hoc comparisons revealed significant differences between each pair of intensity level (S1 vs. S2; S1 vs. S3; S1 vs. S4; S2 vs. S3; S2 vs. S4; S3 vs. S4), in both electrodes. Also we found a significant Intensity × Group interaction [F_(3,306)_ = 2.84; p = 0.038; Cohen’s d = 0.28], with a small effect size (d > 0.20). As may be seen in Table 2, the patients showed slightly larger amplitudes to the intensities S1, S2, and S3, but smaller to the S4, as compared to the healthy controls. However, no significant differences were observed in post-hoc comparisons.

**Table 2:** Left: N1-P2 peak-to-peak amplitudes (in µV) measured at the Fz and Cz electrodes to the different intensities (S1, S2, S3 and S4) (standard deviations in parentheses), for patients with Fibromyalgia (FM) and healthy controls (HC). Right: Repeated measures ANOVA analysis significant results and effect sizes (Cohen’s d).

Stimuli	FM (n = 45)		HC (n = 59)		ANOVA results		
	N1-P2 amplitude		N1-P2 amplitude				
	Fz	Cz	Fz	Cz		F (p)	Cohen’s d
S1 (70 dB)	9.09 (3,96)	10.46 (4.46)	8.53 (3.17)	9.47 (3.24)	Electrode effect	143.11 (< .001)	2.35
S2 (80 dB)	10.78 (4.74)	12.74 (4.92)	9.60 (3.71)	11.05 (3.96)	Intensity effect	141.82 (< .001)	2.35
S3 (90 dB)	10.78 (4.74)	13.87 (4.93)	9.60 (3.71)	12.79 (4.52)	Elecgrode × intensity effect	78.338 (< .001)	1.73
S4 (105 dB)	13.42 (5.93)	17.81 (6.98)	13.91 (5.83)	17.97 (7.23)	Intensity × group effect	2.84 (< .05)	0.28

**Figure 2:**
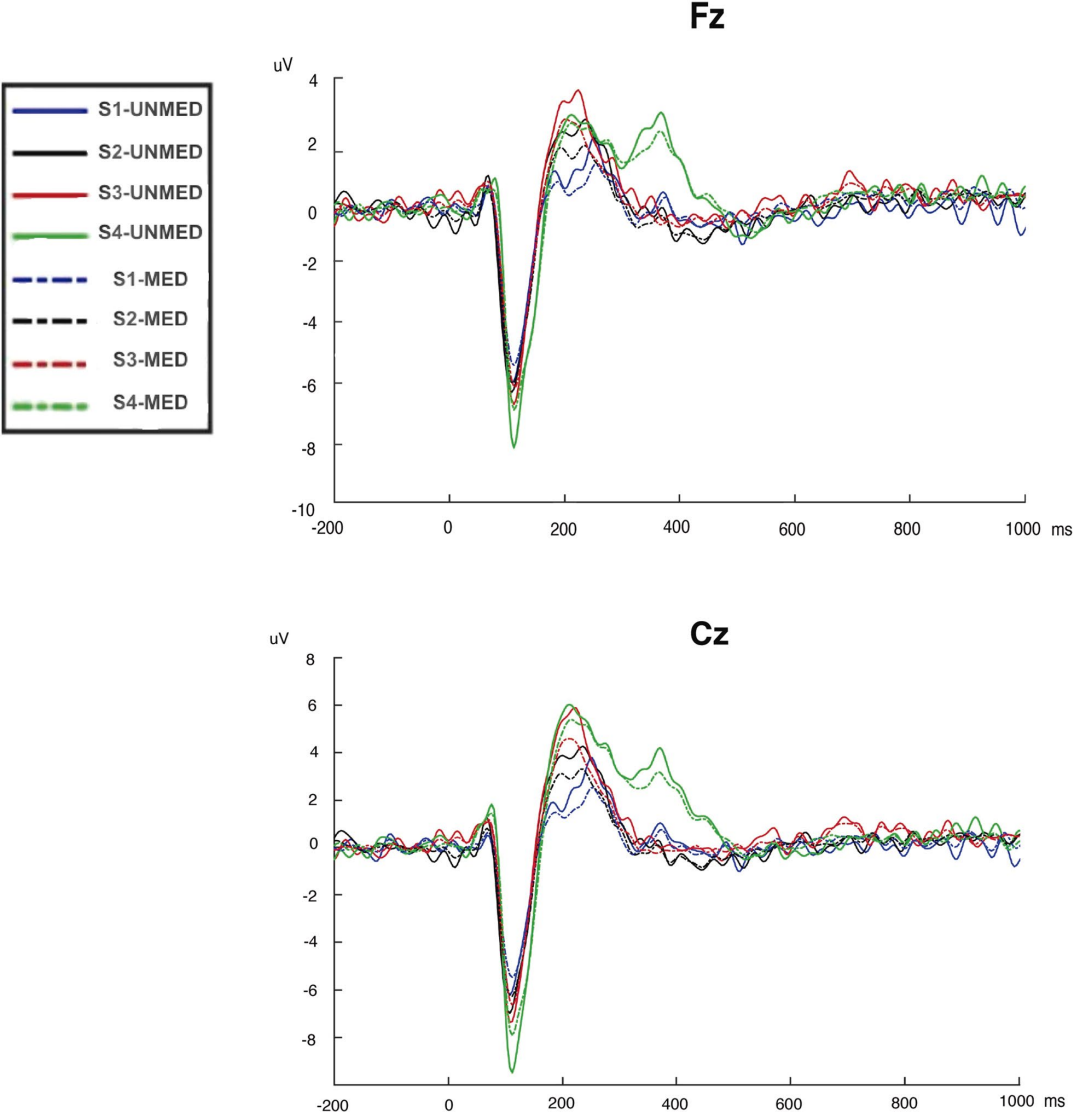
Electrical brain activity in response to auditory stimulation in patients with fibromyalgia without CNS medication (UNMED) or with CNS medication (MED). The waveforms represent the auditory evoked potentials (AEPs) at electrodes Fz (up) and Cz (bottom) elicited by the tones of 70 dB (S1; blue line), 80 dB (S2; black line), 90 dB (S3; red line) and, 105 dB (S4; green line).

We divided the patients sample according to their medication pattern (24 patients using CNS medication; 21 patients non using CNS medication). The ANOVA performed to assess the effect of medication in the FM group revealed significant effects for Electrode [F_(1,43)_ = 84.71; p < 0.001; Cohen’s d = 2.78]; Intensity [F_(3,129)_ = 49.79; p < 0.001; Cohen’s d = 2.12], and Intensity × Electrode [F_(3,129)_ = 31.55; p < 0.001; Cohen’s d = 1.70]. Also, we found a main effect of Group of Medication [F_(1,43)_ = 5.10; p = 0.029; Cohen’s d = 0.66], with a medium effect size (d > 0.5). Post-hoc comparisons revealed that patients using medication with central effects showed smaller N1-P2 amplitudes (mean = 10.98 µV) than patients without medication affecting the CNS (including non-medicated patients or patients using anti-inflamatory or peripheral analgesics; mean N1-P2 amplitude = 13.96 µV), irrespective of the intensity and electrode (see Fig. 2).

Finally, we calculated the intensity/amplitude slope of N1-P2 at Cz (because it was the electrode showing the largest amplitudes) for the patients and correlated it with the clinical variables. No significant correlation was observed with any of the FM symptoms (assessed by VAS, FIQ, SF-36, BDI, PSQI, and pain threshold and tolerance values), irrespective of the patients’ medication pattern.

### Study 2

#### Demographic and clinical variables

As may be seen in Table 3, both groups were comparable in age and level of education. Concerning the clinical variables, patients with FM were overall more affected than controls. Effect size analyses for the clinical variables (VAS, SSS, WPI, BDI and, PSQI) showed a large effect (Cohen’s d > 0.80). The percentage of patients using each type of medication was the following: 33.3% used analgesics, 33.3% NSAIDs, 51.9% anxiolytics, 22.2% opioids, 3.7% migraine medication, 48.1% antidepressants, 14.8% sedatives, and 22.2% antiepileptic drugs (see *Supplementary material*; Supplementary Table S2).

**Table 3:** Mean values (standard deviations in parentheses) of the demographic and clinical variables measured for the patients with fibromyalgia (FM) and healthy controls (HC). Results of the t-tests comparisons and effect sizes (Cohen’s d).

Variable	FM n	FM Mean (SD)	HC n	HC Mean (SD)	t	p	Cohen’s d
Age	26	50.31 (9.20)	27	48.44 (11.18)	0.66	n.s.	–
Years of education	26	9.96 (4.06)	23	11.26 (4.16)	− 1.11	n.s.	–
VAS pain	28	6.24 (1.64)	30	3.23 (3.33)	4.42	< 0.001	1.15
VAS health status	28	6.14 (2.09)	30	3.65 (2.97)	3.70	< 0.01	0.97
VAS mood status	27	5.93 (2.40)	30	3.75 (2.97)	3.03	< 0.01	0.81
VAS non-restorative sleep	28	6.96 (2.28)	30	3.61 (3.22)	4.60	< 0.001	1.20
SSS	25	10.48 (1.16)	29	7.62 (1.95)	6.64	< 0.001	1.78
WPI	25	12.52 (4.32)	29	1.86 (1.98)	11.35	< 0.001	3.17
BDI	27	22.93 (10.78)	23	10.96 (5.60)	6.01	< 0.001	1.39
PSQI total	27	13.48 (5.06)	20	5.70 (3.80)	5.77	< 0.001	1.74

VAS: Visual Analogue Scale; SSS: Symptom Severity Score; WPI: Widespread Pain Index; BDI: Beck Depression Inventory; PSQI: Pittsburgh Sleep Quality Index; n.s.: non-significant.

#### AEPs to tones

**Figure 3:**
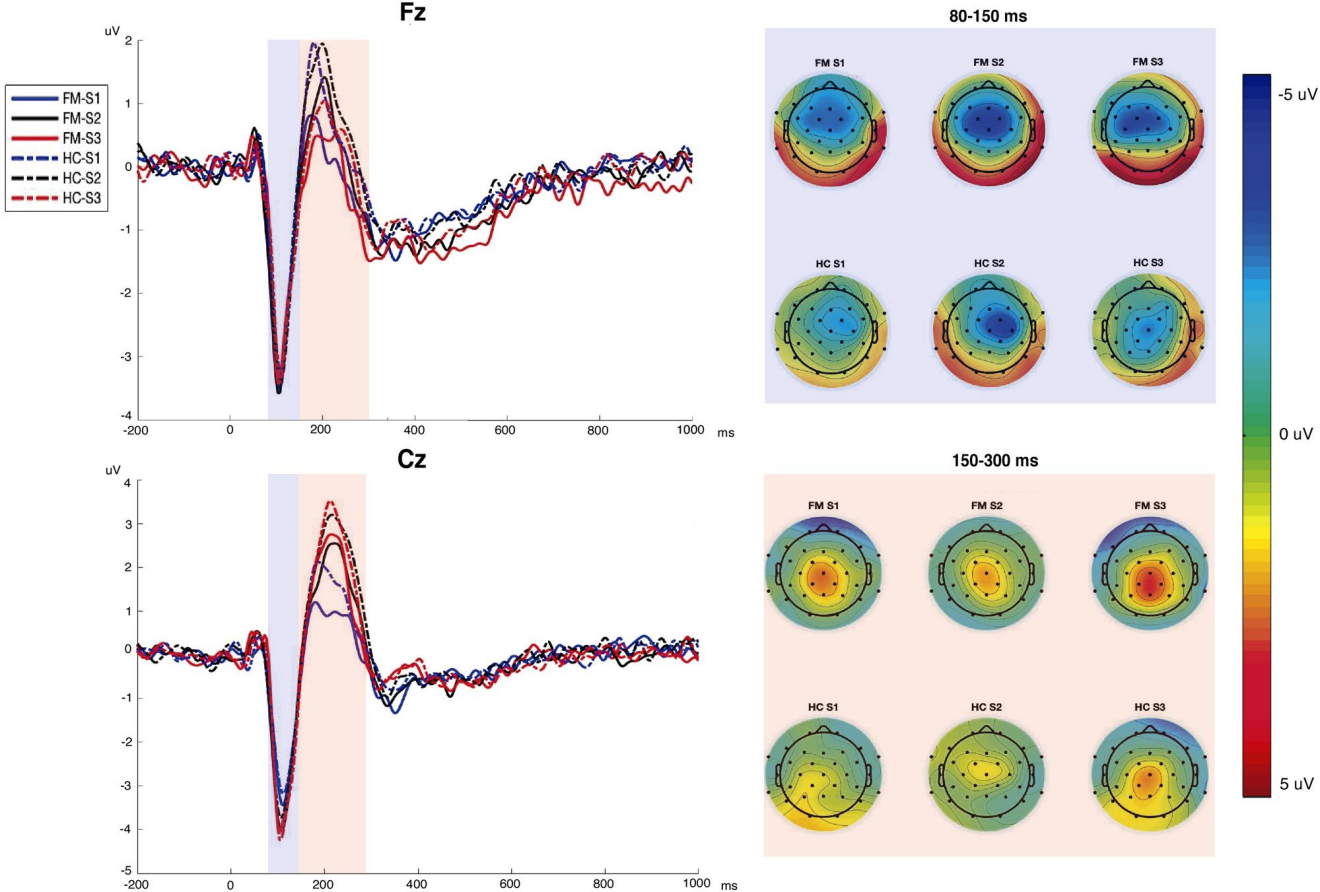
Right. Auditory evoked potentials (AEPs) at electrodes Fz and Cz elicited by the tones of 70 dB (S1; blue line), 90 dB (S2; black line) and, 105 dB (S3; red line) in patients with fibromyalgia (FM) and healthy controls (HC). Left. Scalp distribution of AEP amplitudes elicited by the three stimuli. The upper part shows the topography of N1 (measured as the mean value from 80 to 150 ms), and the lower part shows the topography of P2 (measured from 150 to 300 ms).

As may be seen in Fig. 3, in this study (applying a block presentation) we also observed larger N1-P2 amplitudes over Cz than Fz for all the intensities, and similar topographies for N1 and P2 than in study 1. At odds with the previous study, the larger N1-P2 amplitudes were not associated to the loudest tones.

A repeated measures ANOVA on N1-P2 peak-to-peak amplitude showed a significant effect of Electrode [F_(1,54)_ = 34.72; p =  < 0.001; Cohen’s d = 1.59], Intensity [F_(2,108)_ = 20.55; p =  < 0.001; Cohen’s d = 1.24], and Electrode x Intensity [F_(2,108)_ = 18.31; p =  < 0.001; Cohen’s d = 1.15]. Post-hoc comparisons revealed that the N1-P2 amplitudes to the highest intensity (S3) were not significantly larger to those for the lowest intensities (S1 or S2). As may be seen in Table 4, the HC showed slightly larger amplitudes in the intensities towards all the stimuli regardless of the electrode; however, the analysis did not show significant effects of Group.

**Table 4:** Left: Mean N1-P2 amplitude (in µV) in Fz and Cz electrodes for each tone intensity (standard deviation in parentheses), for the patients (FM) and healthy controls (HC). Right. Repeated measures ANOVA analysis significant results and effect sizes (Cohen’s d).

Stimuli	FM (n = 26)		HC (n = 30)		ANOVA results		
	N1-P2 amplitude		N1-P2 amplitude			F (p)	Cohen’s d
	Fz	Cz	Fz	Cz			
S1 (70 dB)	5.41 (2.20)	5.92 (2.18)	5.96 (1.91)	6.45 (2.12)	Electrode effect	34.72 (< 0.001)	1.59
S2 (90 dB)	6.39 (2.87)	7.62 (2.78)	6.75 (2.41)	8.40 (2.70)	Intensity effect	20.55 (< 0.001)	1.24
S3 (105 dB)	5.37 (2.77)	7.48 (2.79)	6.08 (2.45)	8.66 (2.80)	Electrode × intensity effect	18.31 (< 0.001)	1.15

**Figure 4:**
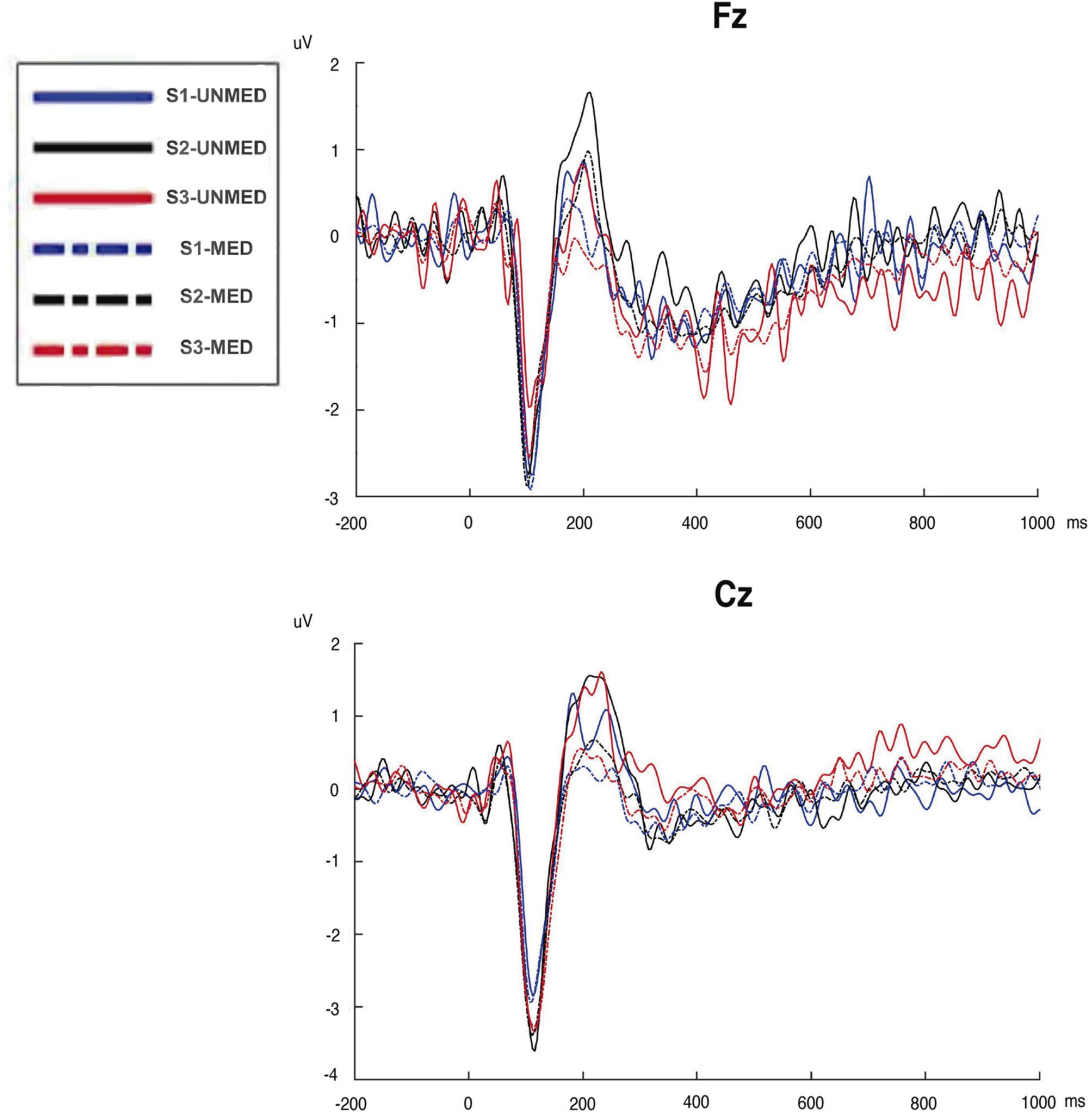
Auditory evoked potentials (AEPs) at electrodes Fz (up) and Cz (down) elicited by the tones of 70 dB (S1; blue line), 90 dB (S2; black line) and, 105 dB (S3; red line) in patients with FM (without medication (UNMED), and with medication of the CNS (MED)).

We divided the sample of patients according to their consumption of medication (17 patients using CNS medication and 8 patients non using CNS medication). The ANOVA showed significant effects for Intensity [F_(2,46)_ = 7.74; p < 0.01; Cohen’s d = 1.15], Electrode [F_(1,23)_ = 10.34; p < 0.01; Cohen’s d = 1.34], and Intensity × Electrode [F_(2,46)_ = 4.05; p = 0.024; Cohen’s d = 0.84]. Although the patients with CNS medication (n = 17) showed smaller N1-P2 amplitudes that patients without it (n = 8; 5.90 µV vs. 7.43 µV), the effect of Group of medication did not reach the significance level [F_(1,23)_ = 3.07; p = 0.093] (see Fig. 4).

Finally, no significant correlation was found between FM symptoms (assessed by VAS, SSS, WPI, BDI, and PSQI) and the amplitude/intensity slope of the N1-P2 complex in Cz for the patients, regardless of their medication pattern.

## Discussion

It is generally assumed that patients with FM present central alterations in the processing of both nociceptive and non-nociceptive stimuli^58^. The hypotheses of generalized hypervigilance, central sensitization, and reduced habituation have been proposed as possible explanatory mechanisms of the FM syndrome^10,17,18,26^. However, the evidence is far from being conclusive. Studies analyzing brain activity when processing somatosensory stimulation found evidence in favor of those pathophysiological mechanisms^13–18^. Nevertheless, in the auditory modality the presence of augmented processing is not always observed. To provide clarity in this field, we carried out two studies recording auditory evoked potentials (AEPs) elicited by tones of various intensities in women diagnosed with FM and healthy controls, using different stimuli sequences. The peak-to-peak amplitude of the N1-P2 complex, a reliable index of the amount of neural resources devoted to the processing of auditory stimuli^35,59^, was evaluated in both studies. In addition, the effect of medication and the possible relationship between the amplitude/intensity function of the AEPs and the symptomatic picture of the patients were analyzed. Contrary to our hypothesis, we did not find a generalized pattern of sensory amplification in the patients, irrespective of the stimuli sequence used.

Since bottom-up sensory amplification of nociceptive input may be one cause of the FM symptoms^60^, in study 1 we assessed the processing of auditory stimuli when bottom-up mechanisms were engaged. To this end, we used a random presentation of stimuli, where the loudest tones involuntarily capture the participants’ attention^32^. Given the hypervigilance -generalized to all kinds of sensory information- assumed for fibromyalgia^26^, we expected patients to have higher N1-P2 amplitudes in response to the tones, specially to the most intense ones. Although we found a significant interaction between Group and Intensity (interestingly, with smaller AEPs to the more intense tones in the patients), group differences did not reach significance in the post-hoc comparisons. Therefore, our results do not support the presence of increased processing of auditory stimuli in the patients. In addition, the data do not show evidence of alterations in the automatic assignment of attentional resources to the tones in the patients.

In study 2, we used a presentation in blocks of stimuli of the same intensity to study the effect of anticipatory/preparatory attention on auditory processing. As the most intense tones were predictable, the subjects could anticipate their appearance and put into operation their sensory input inhibitory mechanisms to attenuate their impact. Given that previous studies have found deficits in sensory inhibition in FM, either using somatosensory noxious stimuli^2–6^ or annoying high intensity auditory stimulation^19,22^, we expected larger AEPs in the patients, specially in response to the loudest tones. Nevertheless, the findings showed similar brain responses in patients and controls: both groups seem to retain the integrity of their inhibition mechanisms and show a reduction in AEPs amplitude to the 105 dB tones. Thus, although the patients normally report hypersensitivity to noise^19,20,24^, our results from study 2 do not support either the presence of increased processing of auditory stimuli.

This pattern of results is in line with previous studies questioning the generalizability of increased processing and hypervigilance to the auditory modality^14,15,23,24^ but disagrees with previous reports of increased AEPs to auditory tones in patients with FM^19,21,26^. In particular, our study could not replicate the results by Carrillo-de-la-Peña et al.^22^, who also used a block presentation and found increased N1-P2 responses to the 105 dB tones in FM patients. Comparing that paper with the present one, we detected a fundamental difference in relation to the patients' medication: they had a wash-out period of 2 weeks while here patients were not asked to discontinue their medication.

In animal models, it has been proposed that the intensity dependence of AEPs may be an index of serotonergic function^36–38^. That system can be altered by medication with 5-HT effects, which is commonly used by patients with FM. To clarify this issue, we performed additional analyses, using only the FM group. We found that patients with medication affecting the CNS (mainly antidepressants, anxiolytics, analgesics, and anticonvulsants) showed an overall reduction in N1-P2 amplitudes. Similarly, previous literature showed a reduced slope of N1-P2 after antidepressant treatment in healthy volunteers^34,61,62^.

Previous literature in FM found that serotonin levels are related to central sensitization and impaired descending inhibitory pathways^63^. Taking into account the hypothesized relationship between the intensity dependence of the AEPs and serotonin levels^36–38^, and the relationship between serotonin function and pain inhibition^39,40^, we expected to find significant correlations between the N1-P2 amplitude/intensity slope and fibromyalgia symptoms’ severity. However, as also reported by Triñanes et al.^64^, we did not found a significant relation. This lack of correlation between AEPs slope and FM severity could be explained by the effect of medication, since patients with more severe symptoms likely take more medication, which in turn may reduce AEPs’ amplitude.

One of the main limitations of the present study is the lack of control of medication used by patients with FM. Although it would be desirable to have a wash-out period, this measure is not free from criticisms. First, it is questionable to ask patients discontinue treatment when pain and the other symptoms are very severe, what could result in a selection of the mild severity patients. In addition, a wash-out period could produce withdrawal adverse effects (drug dependence), also difficult to control. In any case, the medication effects observed in this report should be taken into account in the design and analyses of future studies. Another possible limitation is the lack of a subjective evaluation of the tones by the participants. Since many studies report the presence of subjective auditory hypersensitivity in FM^19–21^, it would be interesting to include the patients’ subjective assessment of intensity and unpleasantness of the presented stimuli. In order to obtain a more homogeneous sample, we only used women, what limits generalization of results to men. Moreover, since the age-of-onset of menopause modulates pain and non-pain sensitivity in women with FM^65^, it would be interesting to take into account the early transition to menopause of the participants in future research.

## Conclusions

As a conclusion, using a large sample of participants, we could not replicate previous reports of impaired inhibitory modulation of high intensity tones in patients with FM, independently of the attentional mechanism engaged in the performance of the task. Altough this pattern of findings may challenge the hypothesis of the hypersensitivity/hypervigilance in the auditory modality, given the observed effects of medication, we could not discard it as an etiopathological mechanism for fibromyalgia. Future studies on the brain activity of FM patients should take into account the critical role of pharmacological treatments with central effects. Altogether, these findings are relevant for the search of objective biomarkers and explanatory theories for fibromyalgia using event-related potentials.

## Supplementary Information

Supplementary Information.

## Data Availability

The datasets used and/or analysed during the current study are available from the corresponding author on reasonable request.
