# Supplementary material for: Effects of intensity, attention and medication on auditory-evoked potentials in patients with fibromyalgia
Source: Sci Rep. 2020 Dec 14;10:21904. doi: 10.1038/s41598-020-78377-0 (PMC7736365; doi:10.1038/s41598-020-78377-0)
Supplement: Supplementary file 1 — Supplementary Information. [file 41598_2020_78377_MOESM1_ESM.docx]

**Title:** **Effects of intensity, attention and medication on auditory-evoked potentials in patients with fibromyalgia.**

**Authors:** Samartin-Veiga, N.*^1^, MSc; González-Villar, AJ.^1,2^, PhD; Triñanes, Y.^1^ MSc; Gómez-Perretta, C. ^†3^, MD & Carrillo-de-la-Peña, MT.^1^, PhD

^1^ Departamento de Psicoloxía Clínica e Psicobioloxía. Facultade de Psicoloxía. Universidade de Santiago de Compostela. Santiago de Compostela. Spain.

^2^ Psychological Neuroscience Lab, Psychology Research Centre, School of Psychology, University of Minho, Braga, Portugal.

^3^ Research Foundation of La Fe Hospital, Valencia, Spain.

**SUPPLEMENTARY MATERIAL**

**Supplementary Table S1.** Medication pattern of the participants with fibromyalgia in Study 1 and its classification in central nervous system (CNS) medicated or unmedicated.

| Study 1 |  |  |
| --- | --- | --- |
| Participant | **CNS-med (2)**  **CNS-unmed (1)** | **Drugs** |
| 1 | 1 | NSAIDs |
| 2 | 1 | none |
| 3 | 2 | antidepressant |
| 4 | 2 | anxiolytics and antidepressants |
| 5 | 1 | none |
| 6 | 2 | anxiolytics |
| 7 | 1 | NSAIDs |
| 8 | 1 | NSAIDs |
| 9 | 2 | antiepileptic |
| 10 | 2 | antidepressant |
| 11 | 2 | antidepressant |
| 12 | 2 | anxiolytics and antidepressants |
| 13 | 1 | none |
| 14 | 2 | antidepressant |
| 15 | 1 | none |
| 16 | 1 | none |
| 17 | 1 | NSAIDs |
| 18 | 1 | none |
| 19 | 1 | none |
| 20 | 1 | none |
| 21 | 1 | NSAIDs |
| 22 | 2 | anxiolytics |
| 23 | 2 | anxiolytics and antidepressants |
| 24 | 2 | anxiolytics |
| 25 | 1 | NSAIDs |
| 26 | 2 | antidepressant |
| 27 | 2 | anxiolytics |
| 28 | 1 | NSAIDs |
| 29 | 1 | none |
| 30 | 1 | none |
| 31 | 1 | none |
| 32 | 1 | NSAIDs |
| 33 | 2 | anxiolytics and antidepressants |
| 34 | 1 | none |
| 35 | 1 | NSAIDs |
| 36 | 2 | antidepressant |
| 37 | 2 | anxiolytics |
| 38 | 2 | antidepressant |
| 39 | 2 | NSAIDs and antidepressants |
| 40 | 2 | anxiolytics |
| 41 | 2 | anxiolytics |
| 42 | 1 | none |
| 43 | 2 | NSAIDs and antidepressants |
| 44 | 2 | anxiolytics |
| 45 | 2 | anxiolytics |
| 46 | 2 | antiepileptic |
| 47 | 2 | anxiolytics and antidepressants |
| 48 | 1 | none |
| 49 | 2 | NSAIDs and antidepressants |
| 50 | 2 | antiepileptic |

**Supplementary Table S2.** Medication pattern of the participants with fibromyalgia in Study 2, and its classification in central nervous system (CNS) medicated or unmedicated.

| Study 2 |  |  |
| --- | --- | --- |
| Participant | CNS-med (2)  CNS-unmed (1) | Drugs |
| 1 | 1 | none |
| 2 | 2 | analgesic |
| 3 | 1 | none |
| 4 | 2 | Opioid, antiepileptic, antidepressant and anxiolytic |
| 5 | 1 | none |
| 6 | 2 | Antidepressant, anxiolytic and hypnotic/sedative |
| 7 | 2 | Antidepressant, anti-migraine and NSAIDs |
| 8 | 1 | none |
| 9 | 2 | Opioid, antiepileptic, antidepressant and NSAIDs |
| 10 | 2 | Analgesic, NSAID, antiepileptic, antidepressant and anxiolytic |
| 11 | 1 | none |
| 12 | 2 | antiepileptic, dopa agent, anxiolytic, hypnotic, NSAIDs and analgesic |
| 13 | 2 | Antidepressants, anxiolytic and hypnotic |
| 14 | 2 | NSAIDs and antiepileptic |
| 15 | 2 | Opioids, NSAIDs, anxiolytic and antidepressant |
| 16 | 2 | antidepressant, anxiolytic and opioids |
| 17 | 2 | Opioid, antidepressant, anxiolytic and analgesic |
| 18 | 2 | Anxiolytic and NSAIDs |
| 19 | 2 | Analgesic, opioid and NSAIDs |
| 20 | 1 | none |
| 21 | 2 | Anxiolytic, analgesic and antidepressant |
| 22 | 1 | analgesic |
| 23 | 2 | analgesic and NSAIDs |
| 24 | 2 | Antidepressant, anxiolytic and antiepileptic |
| 25 | 1 | none |
| 26 | 2 | Anxiolytic and analgesic |
| 27 | 2 | Anxiolytic, hypnotic/sedative, antidepressants, NSAIDs, opioid |
